# The Prognostic Value of PERK in Cancer and Its Relationship With Immune Cell Infiltration

**DOI:** 10.3389/fmolb.2021.648752

**Published:** 2021-04-16

**Authors:** Peng Wang, Liying Han, Moxin Yu, Zhengyu Cao, Xiaoning Li, Yunxia Shao, Guoping Zhu

**Affiliations:** ^1^Anhui Provincial Key Laboratory of Molecular Enzymology and Mechanism of Major Diseases, Key Laboratory of Biomedicine in Gene Diseases, Health of Anhui Higher Education Institutes, Anhui Normal University, Wuhu, China; ^2^Department of Clinical Laboratory, Yijishan Hospital of Wannan Medical College, Wuhu, China; ^3^Department of Nephrology, Wuhu Hospital Affiliated to East China Normal University, Wuhu, China

**Keywords:** cancer, biomarker, prognosis, immune cell infiltration, PERK

## Abstract

**Background:** Protein kinase R (PKR)-like endoplasmic reticulum kinase (PERK) is a type I transmembrane protein that functions as an endoplasmic reticulum (ER) stress sensor to regulate global protein synthesis. Recent research studies suggest that PERK, as an important receptor protein of unfolded protein response, is involved in the pathogenesis of many cancers. This study aimed to investigate PERK expression and its relationship with prognosis in pan-cancer and attempted to explore the relevant mechanism of PERK involved in the regulation of cancer pathogenesis.

**Methods:** The Oncomine and TIMER databases were used to analyze the expression of PERK between pan-cancer samples and normal samples. Survival analysis was performed using the PrognoScan, Kaplan–Meier (K-M) plotter, and UALCAN databases. Gene set enrichment analysis (GSEA) was used to perform the functional enrichment analysis of the PERK gene in breast invasive carcinoma (BRCA), head and neck squamous cell carcinoma (HNSC), and thyroid carcinoma (THCA). The TIMER database was used to investigate the correlation between PERK expression and tumor-infiltrating immune cells and analyze the relationship of PERK with marker genes of immune cells which were downloaded from the CellMarker database in BRCA, HNSC, and THCA.

**Results:** PERK was differentially expressed in various cancers, such as breast cancer, liver cancer, lung cancer, gastric carcinoma, lymphoma, thyroid cancer, leukemia, and head and neck squamous cell carcinomas. The high expression of PERK was associated with a poor prognosis in KIRP, LGG, BRCA, and THCA and with a favorable prognosis in HNSC. The results of GSEA indicated that PERK was mainly enriched in immune-related signaling pathways in BRCA, HNSC, and THCA. Moreover, PERK expression was significant positively correlated with infiltrating levels of macrophages and dendritic cells and was strongly associated with a variety of immune markers, especially macrophage mannose receptor 1 (MRC1, also called CD206) and T-helper cells (Th).

**Conclusion:** The high expression of PERK could promote the infiltration of multiple immune cells in the tumor microenvironment and could deteriorate the outcomes of patients with breast and thyroid cancers, suggesting that PERK as well as tumor-infiltrating immune cells could be taken as potential biomarkers of prognosis.

## Introduction

The endoplasmic reticulum (ER) plays a pivotal role in the synthesis and proper folding of most proteins, including almost all secreted proteins (Oakes and Papa, 2015). When the accumulation of unfolded proteins in the ER exceeds a certain limit due to various intracellular and extracellular stimuli, a signal transduction pathway, called the unfolded protein response (UPR), is initiated to respond to this disturbance in ER proteostasis (Hetz et al., 2015). The ability of cells to perceive ER stress is essential for cell survival under adverse conditions (Tabas and Ron, 2011). UPR reaction is mainly mediated by three primary sensors: protein kinase R (PKR)-like endoplasmic reticulum kinase [PERK, also known as eukaryotic initiation factor 2-alpha kinase 3 (EIF2AK3)], inositol-requiring gene 1 (IRE1), and activating transcription factor 6 (ATF6).

Cancer cells usually invade into surrounding tissues. The conditions in these environments are usually unfavorable (hypoxia, lack of glucose, lactic acidosis, oxidative stress, insufficient amino acid supply, etc.), which will hinder protein folding in the ER (Lee et al., 2003; Ma and Hendershot, 2004; Lee and Hendershot, 2006; Moenner et al., 2007). In addition, many cancer cells have to overcome similar internal stresses, including oncogene activation, increased glycolysis, etc., which may cause overwhelming protein synthesis and a large demand for secretory pathways (Tollefsbol and Cohen, 1990; Ruggero, 2013; Dejeans et al., 2014). Accordingly, many studies have reported the activation of the UPR in various primary human solid tumors, including glioblastoma, rhabdomyosarcoma, and carcinomas of the breast, stomach, esophagus, liver, colon, and pancreas (Fernandez et al., 2000; Shuda et al., 2003; Moenner et al., 2007; Shi et al., 2019; Wang et al., 2019; McCarthy et al., 2020). The elevated UPR tends to alleviate the stress damage and promote cancer cell survival. The UPR pathway can also regulate cell survival by modulating apoptosis. When cells are exposed to prolonged ER stress conditions, UPR signaling will eventually induce cell death (Mori, 2000; Ron and Walter, 2007; Tabas and Ron, 2011).

PERK is a type I ER transmembrane protein containing a stress-sensing luminal domain and a cytosolic kinase domain (Shi et al., 1998; Wang et al., 2018). Under normal conditions, the ER chaperone GRP78/BiP associates with the luminal domain, thus inhibiting its activation. Accumulation of unfolded proteins in the ER triggers GRP78/BiP titration and PERK is then activated (Bertolotti et al., 2000; Su et al., 2008). PERK phosphorylates the eukaryotic initiation factor 2α (eIF2α) at serine-51 (Marciniak et al., 2006). Phosphorylation of eIF2α hinders the assembly of the ribosome and consequently reduces the protein translation. However, translation of certain eIF2α downstream effectors, ATF4, and CAAT/enhancer binding protein (C/EBP) homologous protein (CHOP), which modulate cellular survival pathways, is increased upon ER stress. The other PERK substrate, transcription factor NF-E2-related factor 2 (NRF2), regulates cellular redox potential and contributes to cell adaptation (Cullinan and Diehl, 2004).

PERK functions as a mediator in UPR-related disease in humans, including tumorigenesis and neurodegenerative disorders. PERK has been shown to support tumor growth, metastasis, autophagy, and radiation resistance and was therefore proposed as a future therapy target to overcome therapy failure (Bobrovnikova-Marjon et al., 2010; Avivar-Valderas et al., 2011; Rouschop et al., 2013; Liu et al., 2015; Feng et al., 2017; Salaroglio et al., 2017; Zhang et al., 2018). Small molecule inhibitors were designed to inhibit PERK phosphorylation and its downstream signaling, which had been tested in antitumor treatment but showed severe side effects in preclinical studies (Atkins et al., 2013; Axten et al., 2013; Yu et al., 2015). However, the mechanisms underlying the effects of PERK in tumorigenesis and development need further study.

In addition, the interaction between cancer cells and the immune system plays a significant role in the occurrence, development, and treatment of cancer. The tumor microenvironment (TME) is comprised of interacting cancer and stromal cells. Among them, infiltrating immune cells account for a large proportion (Bindea et al., 2013). Almost all types of immune cells, including B cells, CD8^+^ T cells, CD4^+^ T cells, neutrophils, natural killer (NK) cells, and dendritic cells (DC), are found in the TME (Bindea et al., 2013). Different from the canonical antitumor role played by immune cells, immune infiltration into the TME represents a strategy tumor cells use to avoid being killed (Gajewski et al., 2013; Quail and Joyce, 2013; Topalian et al., 2015). A recent study showed that PERK promotes the functionality of tumor myeloid-derived suppressor cells (MDSCs) through stimulation of NRF2, which restricts the immunostimulatory axis of cytosolic mitochondrial DNA-STING-type I IFN (Mohamed et al., 2020). Immunotherapy targeting interactions between immune cells and tumor cells has been developed in recent years to reactivate adaptive and innate immune systems and create a robust antitumoral immune response. Inhibitors augment T-cell activity by blocking programmed cell death protein 1 (PD-1) and PD-1 ligand (PD-L1) and show remarkable clinical effects (Topalian et al., 2015; Gordon et al., 2017). However, there are still too few potential targets for immunotherapies. It is still necessary to further discover more specific or general immune biomarkers in cancer therapy.

**Figure 1:**
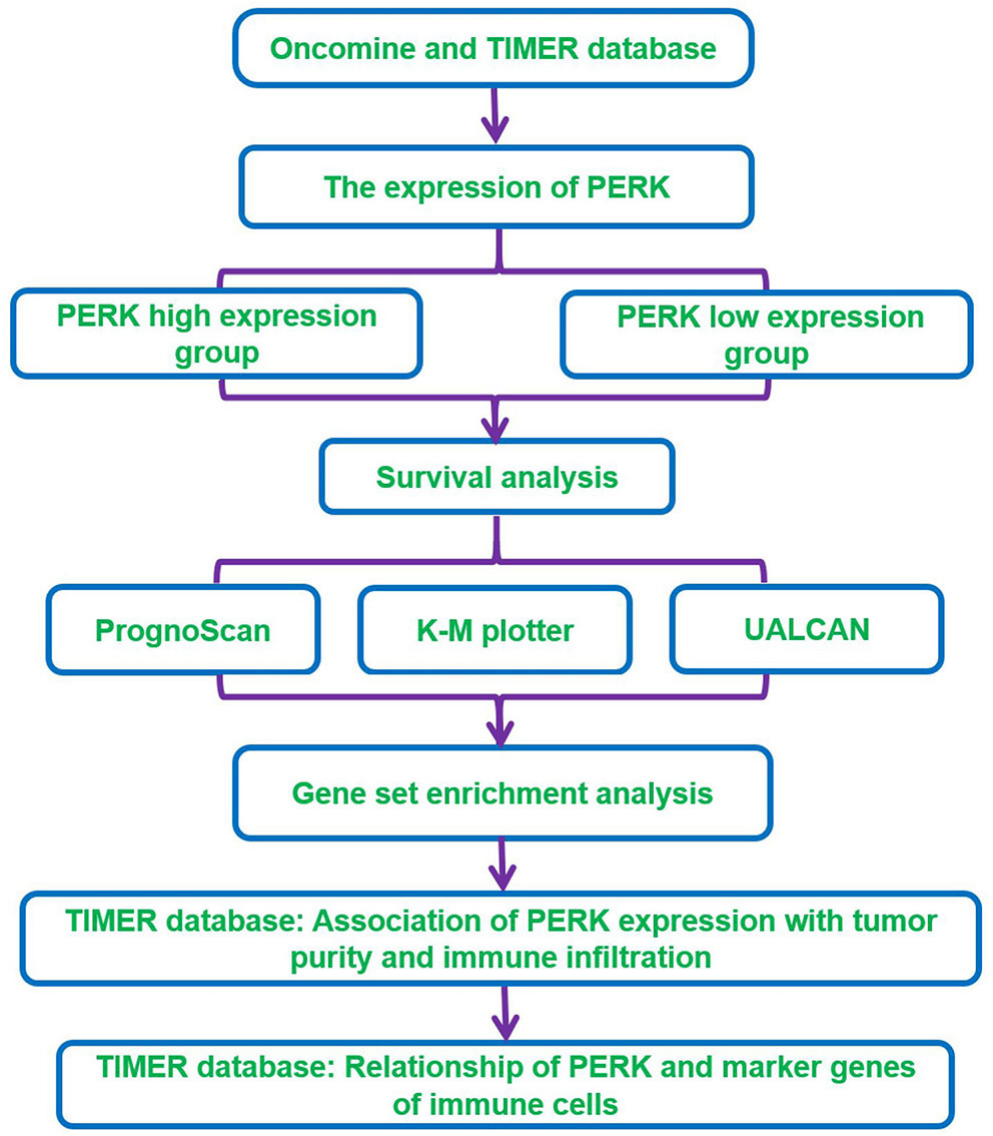
The workflow chart of the study design and analysis.

In this study, we visualized the prognostic landscape of PERK in pan-cancer using databases, including Oncomine, PrognoScan, Kaplan–Meier plotter, and UALCAN. We further investigated the link between PERK and immune cell infiltration of tumors using the Tumor Immunoassay Resource (TIMER). The relationship of PERK with marker genes of immune cells was evaluated by searching the CellMarker database. The workflow of this study design and analysis is summarized in Figure 1. The findings indicate that PERK influences the prognosis of patients with cancers, probably *via* its interaction with infiltrating immune cells.

## Materials and Methods

### Analysis of PERK Expression in Pan-Cancer

The Oncomine database compiled 86,733 samples and 715 gene expression data sets into a single comprehensive database designed to facilitate data mining efforts (Rhodes et al., 2004). TIMER is a database designed for analyzing immune cell infiltrates in multiple cancers. This database employs pathological examination-validated statistical methodology in order to estimate tumor immune infiltration by neutrophils, macrophages, dendritic cells, B cells, and CD4^+^/CD8^+^ T cells (Li et al., 2020). The differential expression of PERK mRNA between pan-cancer samples and normal samples was analyzed by the Oncomine database (https://www.oncomine.org/) and the TIMER database (https://cistrome.shinyapps.io/timer/). The filtering threshold was as follows: |log_2_ fold change (FC)| > 2, *p* value < 0.05, and gene rank with top 10%.

### Survival Analysis

The PrognoScan database is designed to facilitate meta-analyses of gene prognostic value by comparing the relationship between gene expression and relevant outcome including overall survival (OS) in a wide range of published cancer microarray data sets (Mizuno et al., 2009). The Kaplan–Meier plotter is capable to assess the effect of 54,000 genes (mRNA, miRNA, protein) on survival in 21 cancer types including breast (*n* = 6,234), ovarian (*n* = 2,190), lung (*n* = 3,452), and gastric (*n* = 1,440) cancers (Nagy et al., 2018). UALCAN is a comprehensive and interactive web resource for analyzing cancer OMICS data. UALCAN is designed to allow users to identify biomarkers or to perform *in silico* validation of potential genes of interest and perform pan-cancer gene expression analysis (Chandrashekar et al., 2017). The pan-cancer samples were divided into two groups (high-expression group and low-expression group) based on the median of PERK mRNA expression in the PrognoScan database (http://dna00.bio.kyutech.ac.jp/PrognoScan/), Kaplan–Meier plotter database (https://kmplot.com/), and UALCAN database (http://ualcan.path.uab.edu/index.html). The K-M survival curves were utilized to exhibit the overall survival (OS), relapse-free survival (RFS), and distant metastasis-free survival (DMFS) of the two groups.

### Gene Set Enrichment Analysis

Functional enrichment analysis of the PERK gene in breast invasive carcinoma (BRCA), head and neck squamous cell carcinoma (HNSC), and thyroid carcinoma (THCA) was performed using the gene set enrichment analysis (GSEA) software (v4.1.0) (Subramanian et al., 2005). Based on the median expression of the PERK gene, the samples in BRCA, HNSC, and THCA were divided into two groups (high-expression group vs. low-expression group). We conducted enrichment analysis (GO term and KEGG pathway) of the functional gene set defined by GSEA, so as to explore the potential biological pathways that PERK may regulate in BRCA, HNSC, and THCA.

### Analysis of Tumor-Infiltrating Immune Cells

To investigate the correlation between PERK expression and tumor-infiltrating immune cells (B cells, CD8^+^ T cells, CD4^+^ T cells, macrophages, neutrophils, and dendritic cells) in 40 types of cancer, we applied the online tool TIMER (https://cistrome.shinyapps.io/timer/) (Li et al., 2020). *p* < 0.05 was regarded as statistically significant.

### Analysis of the Relationship Between PERK and Immune Cell Marker Genes

The CellMarker database provides a comprehensive and accurate resource of cell markers for various cell types in tissues of human and mouse; 13,605 cell markers of 467 cell types in 158 human tissues/subtissues and 9,148 cell markers of 389 cell types in 81 mouse tissues/subtissues were collected and deposited in CellMarker (Zhang et al., 2019). The 66 marker genes of immune cells, including innate immune cells and acquired immune cells, were downloaded from the CellMarker database (http://biocc.hrbmu.edu.cn/CellMarker/). The correlation of PERK with the marker genes was analyzed in BRCA, HNSC, and THCA using the TIMER database. *p* < 0.05 was considered as statistically significant.

### Statistical Analysis

The independent-samples *t*-test was used for the comparison between two groups. All correlation analyses in this study were performed by the Spearman correlation analysis. *p* < 0.05 was regarded as statistically significant.

## Result

### Expression of PERK in Cancers

**Figure 2:**
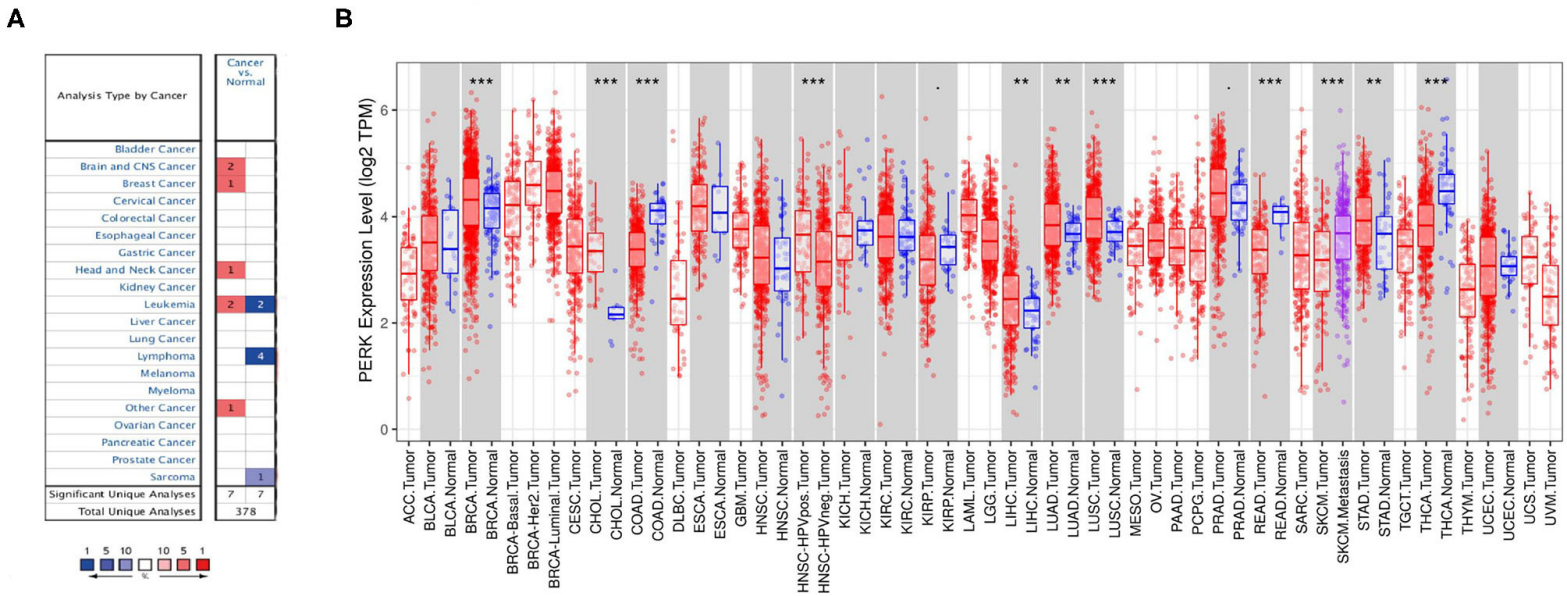
Protein kinase R (PKR)-like endoplasmic reticulum kinase (PERK) expression levels in different types of human cancers. **(A)** Increased or decreased PERK in data sets of different cancers compared with normal tissues in the Oncomine database. **(B)** Human PERK expression levels in different tumor types from the TCGA database were determined by TIMER (*p < 0.05, **p < 0.01, ***p < 0.001).

PERK is a main ER transmembrane sensor which is involved in both integrated stress response (ISR) and UPR (Ron and Walter, 2007; Hetz et al., 2013), which plays a vital role in the occurrence and development of various tumors. To uncover the role of PERK in cancer, we investigated the expression of PERK mRNA between the pan-cancer samples and normal samples through the Oncomine and TIMER databases. As shown in Figure 2A, the red color means that PERK was upregulated in the cancers, and blue means that PERK was downregulated in the cancers. Compared with the normal, the expression of PERK mRNA was upregulated in brain and CNS cancer, breast cancer, head and neck cancer, and other cancers (all *p* < 0.05) and downregulated in lymphoma and sarcoma (all *p* < 0.05) in the Oncomine database. PERK presented a high or low expression in leukemia. The detailed information is listed in Table 1.

**Table 1:** The expression of protein kinase R (PKR)-like endoplasmic reticulum kinase (PERK) mRNA between the pan-cancer samples and normal samples in the Oncomine database.

**Cancer**	**Cancer type**	**Fold change**	***p* value**	**Rank (%)**	**Sample**	**Reference (PMID)**
Brain and CNS cancer	Anaplastic oligoastrocytoma	2.581	1.36E-04	2	10	16357140
Brain and CNS cancer	Glioblastoma	2.112	0.003	8	15	TCGA
Breast cancer	Ductal breast carcinoma *in situ*	4.276	0.019	4	8	16043716
Head and neck cancer	Head and neck squamous cell carcinoma	2.032	4.27E-08	5	54	14729608
Leukemia	B-cell childhood acute lymphoblastic leukemia	2.630	1.17E-39	4	433	20406941
Leukemia	B-cell acute lymphoblastic leukemia	2.360	2.35E-29	4	221	20406941
Leukemia	T-cell childhood acute lymphoblastic leukemia	−8.309	2.20E-35	1	50	21487112
Leukemia	B-cell childhood acute lymphoblastic leukemia	−3.030	9.03E-48	1	242	21487112
Lymphoma	Hodgkin's lymphoma	−2.438	1.08E-09	1	37	18794340
Lymphoma	T-cell/histiocyte-rich large B-cell lymphoma	−2.064	4.49E-05	2	29	18794340
Lymphoma	Unspecified peripheral T-cell lymphoma	−2.032	2.78E-08	7	48	17304354
Lymphoma	Angioimmunoblastic T-cell lymphoma	−2.451	1.35E-04	10	26	17304354
Other cancer	Embryonal carcinoma, NOS	2.197	2.40E-08	4	21	16424014
Sarcoma	Gastrointestinal stromal tumor	−2.156	3.20E-04	9	25	21447720

According to the TIMER database, the expression of PERK mRNA was upregulated in BRCA, CHOL, LIHC, LUAD, LUSC, and STAD (all *p* < 0.01) and downregulated in COAD, READ, and THCA (all *p* < 0.01). Interestingly, the expression of PERK had no significant difference between HNSC and normal, but it was highly expressed in HPV-positive HNSC compared with HPV-negative HNSC (*p* < 0.001). Similarly, the expression of PERK in metastatic SKCM was upregulated compared with SKCM (*p* < 0.001, Figure 2B).

Combining the results of the two databases, PERK has high expression in brain and CNS cancer, breast cancer, cholangiocarcinoma, liver cancer, lung cancer, and gastric carcinoma and has low expression in lymphoma, sarcoma, colorectal cancer, and thyroid cancer. It also presented a differential expression in leukemia, head and neck squamous cell carcinomas, and skin cutaneous melanoma.

### Survival Analysis Based on PrognoScan, K-M Plotter, and UALCAN Databases

A previous study has shown that PERK inhibition by siRNA or GSK2656157 (a small molecule inhibitor against the PERK/elF2α/ATF4 pathway) might improve clinical prognosis and enhance the treatment of esophageal squamous cell carcinoma (ESCC) patients (Wang et al., 2017), but little research is reported in other types of cancers. To widely explore the relationship of PERK expression and prognosis, the PrognoScan, K-M plotter, and UALCAN databases were employed to analyze the change of OS, RFS, and DMFS in the PERK high- and low-expression groups divided by the median in pan-cancer.

**Figure 3:**
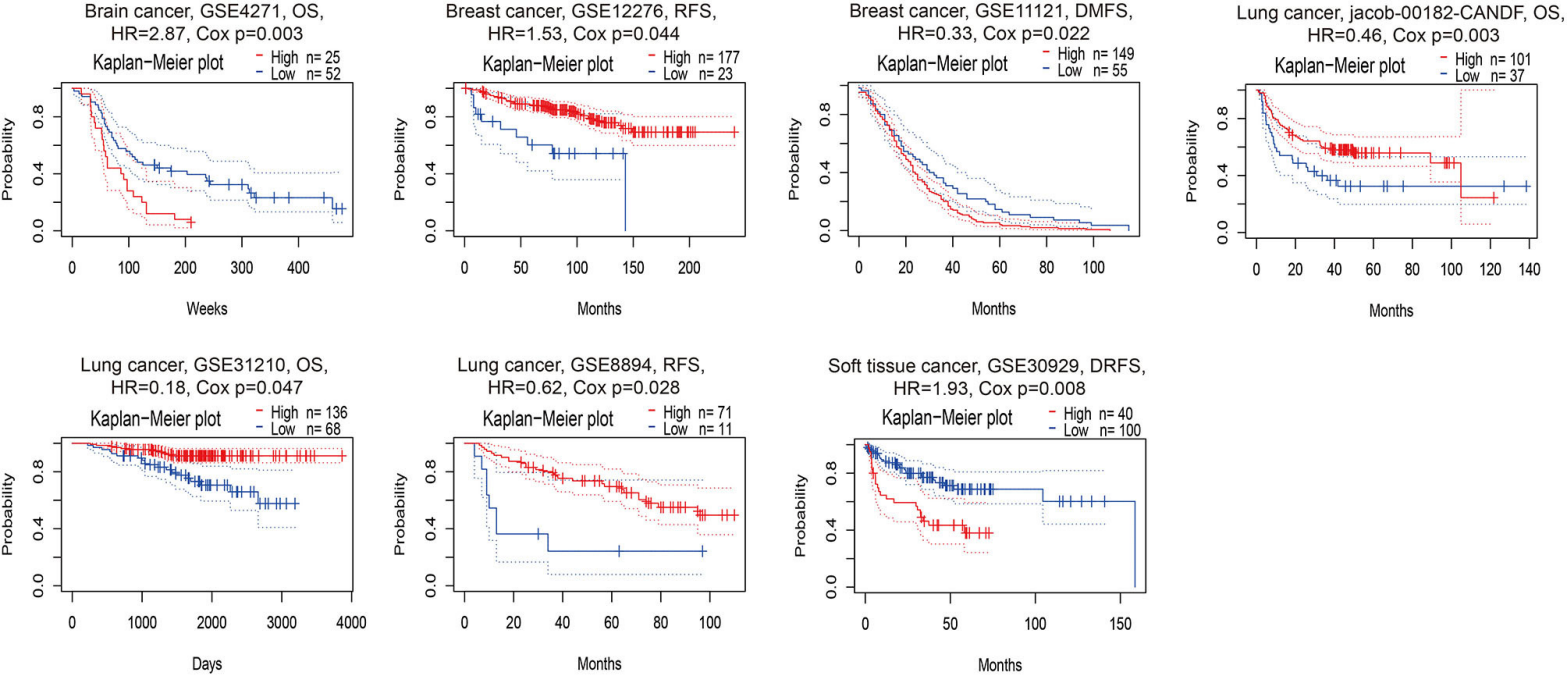
Kaplan–Meier survival curves comparing the high and low expressions of PERK in different cancer types in PrognoScan. DSS, disease-specific survival; OS, overall survival; DMFS, distant metastasis-free survival; DFS, disease-free survival; RFS, relapse-free survival.

According to the PrognoScan database, the high expression of PERK was associated with a poor prognosis in brain cancer (shorter OS, *p* = 0.003) and soft tissue cancer (shorter DRFS, *p* = 0.008) and related to a favorable prognosis in lung cancer (longer OS and RFS, *p* < 0.05, Figure 3). Intriguingly, the high expression of PERK in breast cancer displayed an opposite prognosis with longer RFS (*p* = 0.044) and shorter DMFS (*p* = 0.022, Figure 3) in the absence of OS data. This aroused our interest in whether PERK could be used as a prognostic marker for breast cancer, and we will further study the prognostic value of PERK in The Cancer Genome Atlas (TCGA) database.

**Figure 4:**
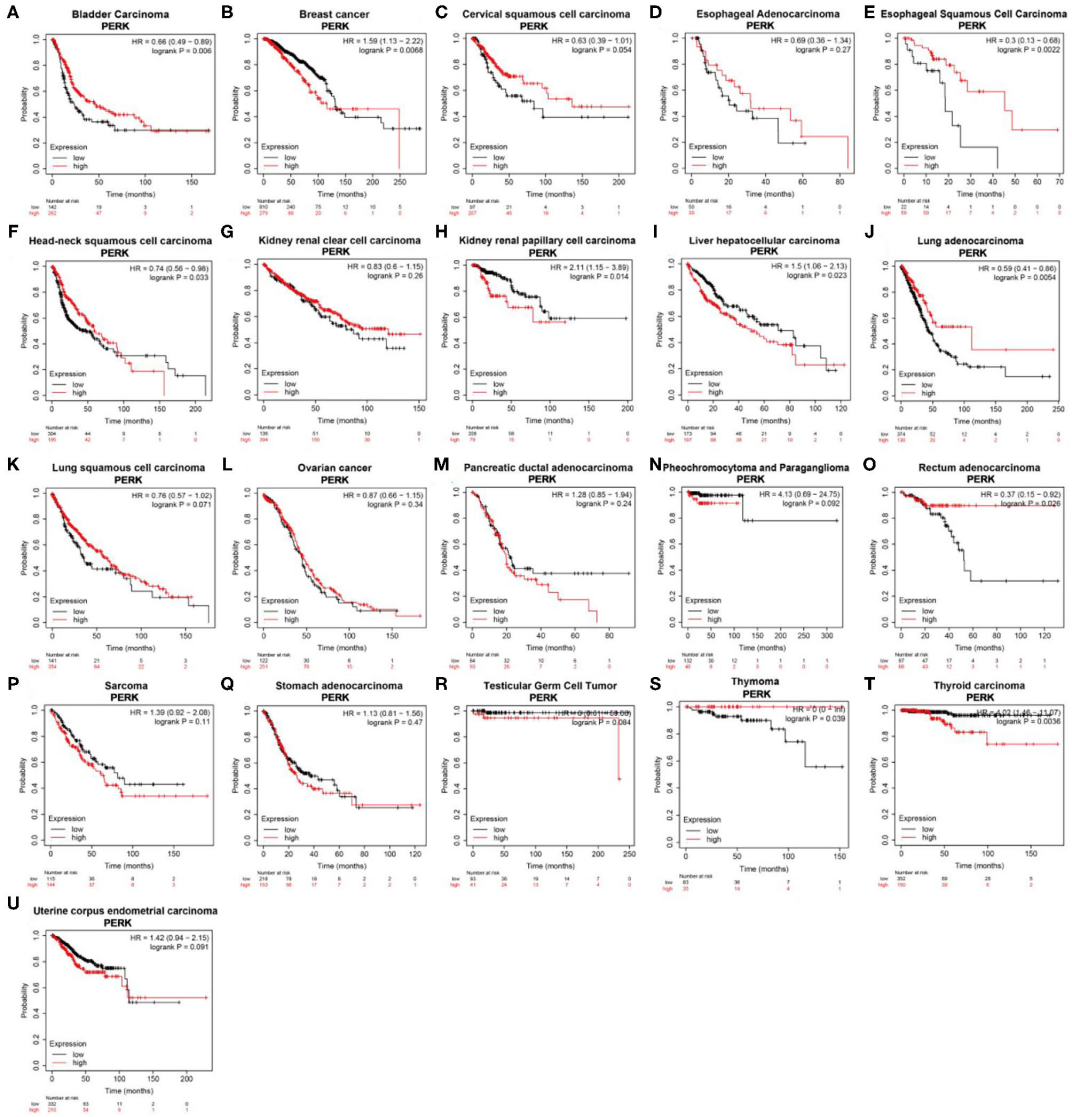
Kaplan–Meier overall survival curves comparing the high and low expressions of PERK in different types of cancer in the Kaplan–Meier plotter.

According to the K-M plotter which is mainly based on Affymetrix microarray data, the survival curve of genes in cancer can be plotted, and pan-cancer data in the TCGA database can also be analyzed (Hou et al., 2017). The high expression of PERK was associated with a favorable prognosis in bladder carcinoma (*p* = 0.006), esophageal squamous cell carcinoma (*p* = 0.0022), lung adenocarcinoma (*p* = 0.0054), rectum adenocarcinoma (*p* = 0.026), and thymoma (*p* = 0.039, Figure 4) and related to a poor prognosis in kidney renal papillary cell carcinoma (*p* = 0.014), liver hepatocellular carcinoma (*p* = 0.023), and thyroid carcinoma (*p* = 0.0036, Figure 4). In particular, PERK in breast cancer seemed to have a poor prognosis (OS < 130 months, *p* = 0.0068, Figure 4). Similar results were obtained in head and neck squamous cell carcinoma, and it had a favorable prognosis (OS < 100 months, *p* = 0.033, Figure 4). PERK expression was not significantly correlated with the prognosis of other cancers, such as cervical squamous cell carcinoma (*p* = 0.054), esophageal adenocarcinoma (*p* = 0.27), kidney renal clear cell carcinoma (*p* = 0.26), lung squamous cell carcinoma (*p* = 0.071), ovarian cancer (*p* = 0.34), pancreatic ductal adenocarcinoma (*p* = 0.24), pheochromocytoma and paraganglioma (*p* = 0.092), sarcoma (*p* = 0.11), stomach adenocarcinoma (*p* = 0.47), testicular germ cell tumor (*p* = 0.084), and uterine corpus endometrial carcinoma (*p* = 0.091, Figure 4).

**Figure 5:**
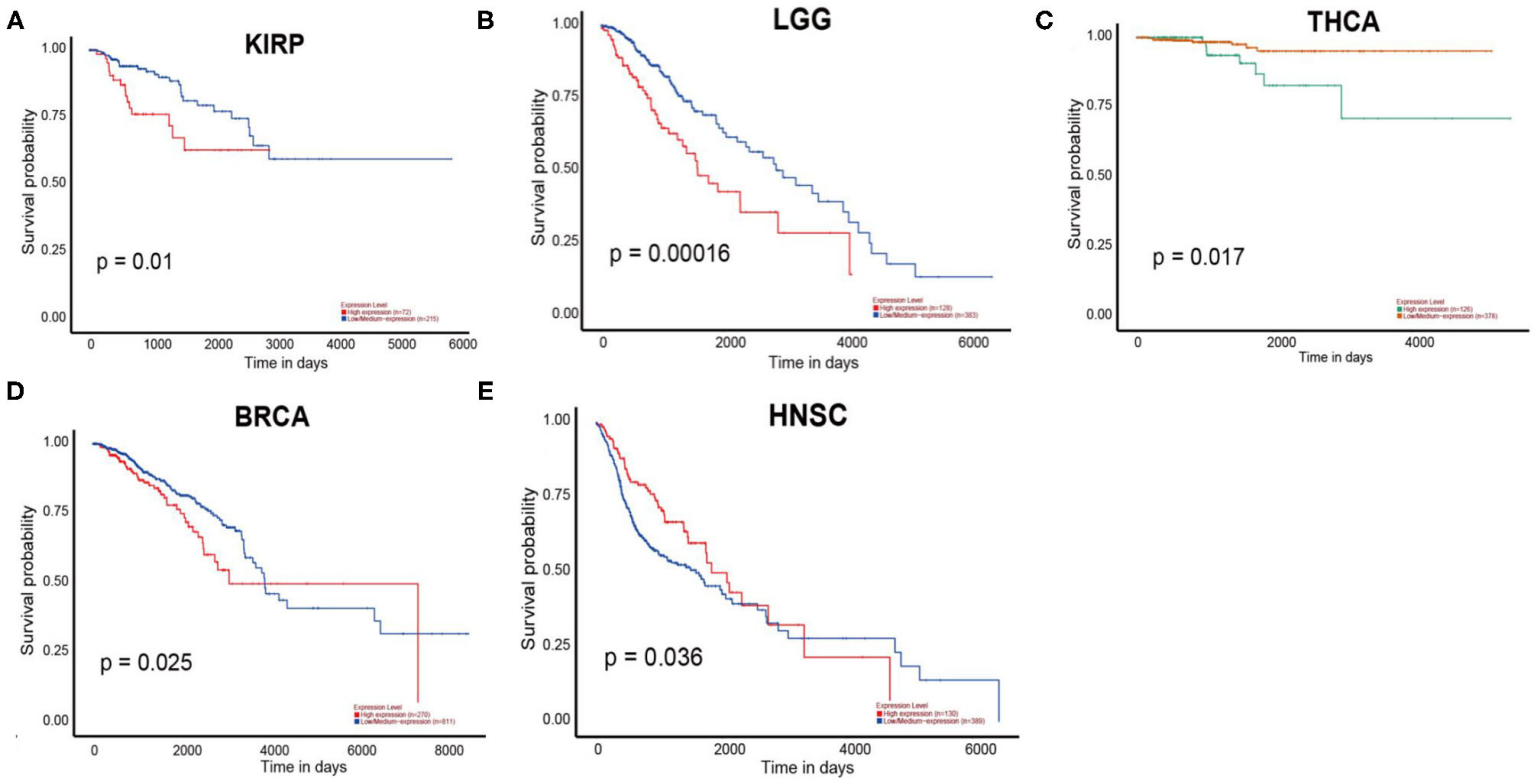
Overall survival curves comparing the high and low expressions of PERK in five different types of cancer in the UALCAN database.

According to the UALCAN database, the high expression of PERK was associated with a poor prognosis in kidney renal papillary cell carcinoma (KIRP) (*p* = 0.01), brain lower grade glioma (LGG) (*p* = 0.00016), and THCA (*p* = 0.017, Figure 5). Consistent with the results of the Kaplan—Meier plotter database, PERK expression in BRCA had a poor prognosis (OS < 4,000 days, ~130 months, *p* = 0.025, Figure 5). Similar results were obtained in HNSC, and it had a favorable prognosis (OS < 3,000 days, ~100 months, *p* = 0.036, Figure 5). There were no significant differences between PERK expression and the prognosis of other cancers (Supplementary Figure 1).

Together, the high expression of PERK is associated with a poor prognosis in KIRP, LGG, BRCA, and THCA and with a favorable prognosis in HNSC. Furthermore, KIRP and LGG were ruled out because the expression of PERK had no significant difference between KIRP and LGG cancers and the matched normal based on the results of the Oncomine and TIMER databases. BRCA, HNSC, and THCA will be included in the subsequent studies.

### GSEA of PERK in BRCA, HNSC, and THCA

**Figure 6:**
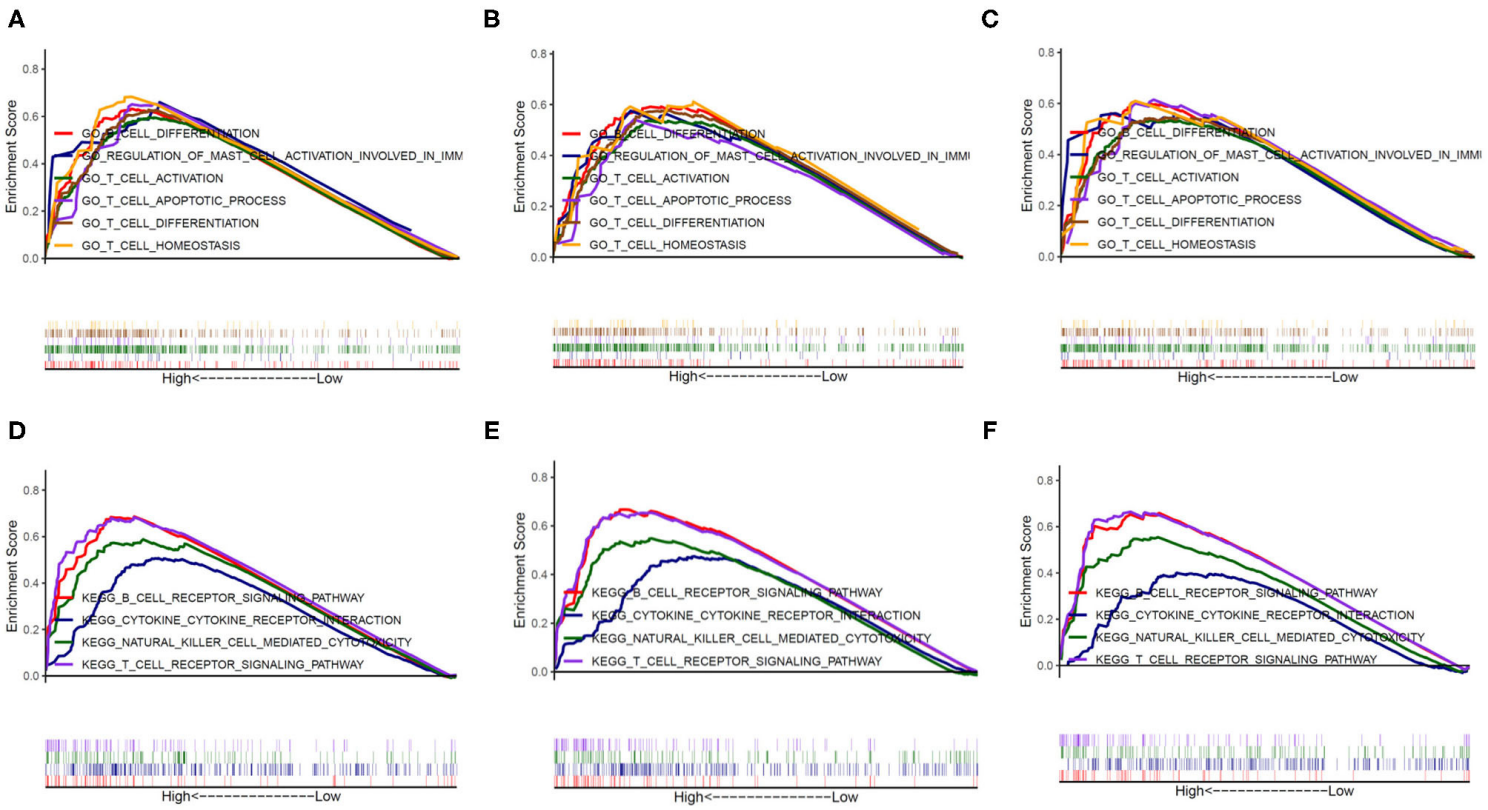
The potential function of PERK analyzed by GESA. The genes of PERK were mainly enriched in six GO terms in BRCA **(A)**, HNSC **(B)**, and THCA **(C)** and enriched in four KEGG terms in BRCA **(D)**, HNSC **(E)**, and THCA **(F)**.

To further investigate the potential functions of PERK in BRCA, HNSC, and THCA, we performed GSEA on the TCGA RNA-seq data. According to the median of PERK expression in the samples of BRCA, HNSC, and THCA, the samples were respectively divided into PERK high-expression group and PERK low-expression group. Ruling out the GO terms (Biological Process) and KEGG terms with *p* < 0.05, it was ranked by the normalized enrichment score (NES). All terms in the low-expression group were excluded, and the terms in the high-expression group were significantly enriched in the immune-related signaling pathways. As shown in Figure 6, the genes of PERK were mainly enriched in the GO terms including the GO_B_CELL_DIFFERENTIATION, GO_T_CELL_DIFFERENTIATION, GO_T_CELL_ACTIVATION, GO_T_CELL_APOPTOTIC_PROCESS, GO_T_CELL_HOMEOSTASIS, and GO_REGULATION_OF_MAST_CELL_ACTIVATION_INVOLVED_IN_IMMUNE_RESPONSE (all *p* < 0.05) and mainly enriched in the KEGG terms including the KEGG_T_CELL_RECEPTOR_SIGNALING_PATHWAY, KEGG_B_CELL_RECEPTOR_SIGNALING_PATHWAY, and KEGG_NATURAL_KILLER_CELL_MEDIATED_CYTOTOXICITY (all *p* < 0.05) in BRCA, HNSC, and THCA. The detailed terms with the high- and low-expression groups are displayed as Supplementary Table 1. These results suggested that PERK might have a close relationship with immunity, especially in BRCA, HNSC, and THCA.

### Association of PERK Expression With Tumor Purity and Immune Infiltration

The tumor microenvironment consists of tumor cells, stromal cells, and infiltrating immune cells (Kim and Bae, 2016). We utilized the TIMER database to explore potential associations between the expression of PERK gene and both tumor purity and infiltration of immune cells in pan-cancer. Interestingly, PERK was significantly associated with tumor purity in a few types of cancer (10/39, *p* < 0.05), and 3 out of 10 cancers showed a negative correlation between PERK and prognosis (*p* < 0.05). Moreover, strong associations were observed between the PERK gene and the infiltrating immune cells, especially B cells (29/39), CD8^+^ T cells (24/39), macrophages (28/39), neutrophils (10/39), and dendritic cells (26/39, all *p* < 0.05, Supplementary Figure 2).

**Figure 7:**
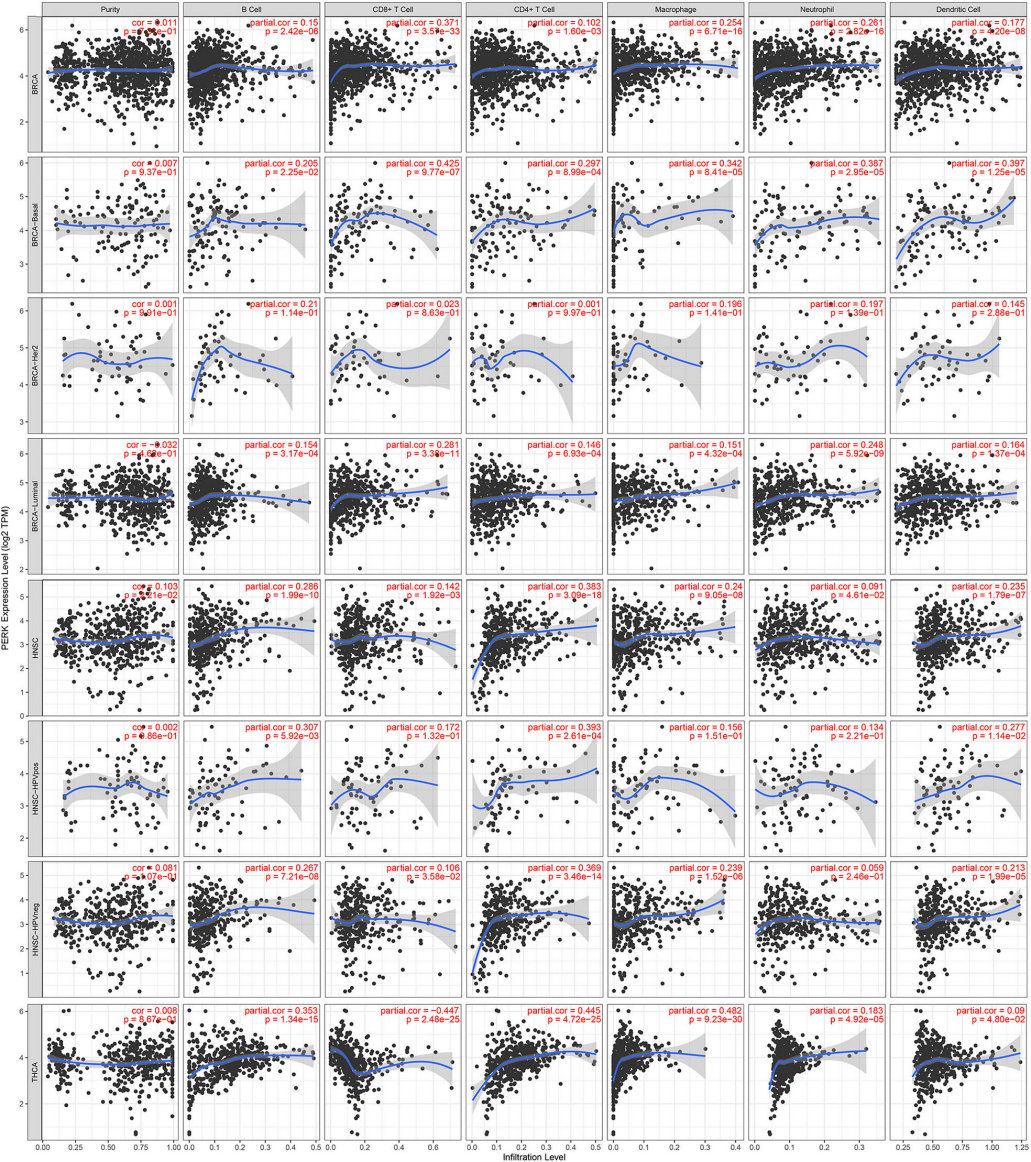
Correlations of PERK expression with immune infiltration level in BRCA, BRCA-basal, BRCA-Her2, BRCA-luminal, HNSC, HNSC-HPV positive, HNSC-HPV negative, and THCA.

Besides, it was worth mentioning that PERK had no significant correlation with the tumor purity of BRCA, but it had a significant correlation with the infiltrating immune cells of BRCA-basal (positive, *p* < 0.05) and BRCA-luminal (negative, *p* < 0.05), but not BRCA-Her2 (Figure 7). Moreover, PERK had a significant positive correlation with the tumor purity of HNSC (*p* < 0.05) rather than HNSC-HPV positive and HNSC-HPV negative. PERK was significantly positively associated with the infiltrating immune cells of HNSC (*p* < 0.05), but CD8^+^ T cells, macrophages, and neutrophils in HNSC-HPV positive and the neutrophils in HNSC-HPV negative were not significantly correlated with PERK (Figure 7). Similar results were shown in BRCA: PERK had no significant correlation with the tumor purity of THCA, but it had a significant correlation with the infiltrating immune cells (*p* < 0.05, Figure 7). Based on the findings from the TIMER database, we proposed that PERK is mainly expressed in immune cells rather than cancer cells, and its function relates to immunological regulation of the tumor microenvironment.

### Relationship of PERK and Marker Genes of Immune Cells in BRCA, HNSC, and THCA

To further explore the effect of PERK expression on tumor-infiltrating immune cells, we analyzed the relationships between PERK expression and various markers of immune cells including innate immune cells and adaptive immune cells in BRCA, HNSC, and THCA *via* the CellMarker database. As shown in Table 2, for the innate immune cells, we observed a significant correlation after adjustment for purity between PERK and some of the markers of macrophages in BRCA, HNSC, and THCA, including INOS and CXCL10 of M1 macrophages and CD163 and IL10 of M2 macrophages (*p* < 0.05). Besides, PERK was also associated with the markers of monocytes in BRCA (*p* < 0.05), neutrophils in HNSC (*p* < 0.05), and dendritic cells in THCA (*p* < 0.05).

**Table 2:** Correlation analysis between PERK and related gene markers of innate immune cells in TIMER in BRCA, HNSC, and THCA.

**Description**	**Gene markers**	**BRCA (*****n*** **=** **1093)**				**HNSC (*****n*** **=** **520)**				**THCA (*****n*** **=** **501)**			
		**Purity**		**None**		**Purity**		**None**		**Purity**		**None**	
		**Cor**	***p***	**Cor**	***P***	**Cor**	***p***	**Cor**	***p***	**Cor**	***p***	**Cor**	***P***
Monocyte	CD14	−0.09	4.65E-03	−0.09	2.88E-03	0.029	5.19E-01	0	9.98E-01	−0.186	3.41E-05	−0.192	1.24E-05
Monocyte	CD86	0.207	4.60E-11	0.171	1.21E-08	0.233	1.82E-07	0.194	8.38E-06	−0.105	1.99E-02	−0.091	3.93E-02
Monocyte	CD16 (FCGR3A)	0.269	6.63E-18	0.25	3.49E-17	0.173	1.13E-04	0.136	1.83E-03	−0.019	6.75E-01	−0.009	8.32E-01
TAM	CD68	0.179	1.26E-08	0.149	7.32E-07	0.271	1.06E-09	0.242	2.39E-08	−0.027	5.50E-01	−0.013	7.71E-01
TAM	CCL2	0.034	2.88E-01	0.007	8.14E-01	0.336	2.01E-14	0.302	2.28E-12	−0.101	2.64E-02	−0.085	5.64E-02
TAM	CCL5	−0.049	1.19E-01	−0.076	1.20E-02	−0.043	3.39E-01	−0.063	1.53E-01	−0.201	7.57E-06	−0.2	5.15E-06
M1 macrophage	INOS (NOS2)	0.118	2.00E-04	0.112	2.08E-04	0.53	6.31E-37	0.537	2.88E-40	0.222	6.94E-07	0.218	7.13E-07
M1 macrophage	CXCL10	0.102	1.26E-03	0.065	3.03E-02	−0.162	3.21E-04	−0.179	4.02E-05	−0.09	4.73E-02	−0.067	1.31E-01
M1 macrophage	TNF-α (TNF)	−0.002	9.54E-01	−0.018	5.41E-01	0.143	1.52E-03	0.116	7.91E-03	−0.167	2.12E-04	−0.154	5.10E-04
M2 macrophage	CD206 (MRC1)	0.303	1.62E-22	0.249	5.55E-17	0.305	4.80E-12	0.231	9.51E-08	−0.015	7.47E-01	−0.009	8.39E-01
M2 macrophage	CD163	0.265	2.06E-17	0.236	1.99E-15	0.279	3.09E-10	0.227	1.61E-07	0.164	2.71E-04	0.17	1.18E-04
M2 macrophage	IL10	0.212	1.32E-11	0.176	4.26E-09	0.274	6.79E-10	0.222	3.04E-07	0.085	6.08E-02	0.084	5.92E-02
Neutrophils	CD66b (CEACAM8)	0.011	7.30E-01	0.026	3.95E-01	0.179	6.73E-05	0.182	2.78E-05	−0.073	1.06E-01	−0.068	1.27E-01
Neutrophils	CD11b (ITGAM)	0.185	3.89E-09	0.172	1.02E-08	0.393	1.24E-19	0.374	8.36E-19	−0.087	5.48E-02	−0.073	9.91E-02
Neutrophils	CCR7	0.032	3.18E-01	−0.005	8.76E-01	0.334	2.63E-14	0.286	2.56E-11	−0.043	3.38E-01	−0.031	4.80E-01
Neutrophils	CD15 (FUT4)	0.244	6.85E-15	0.202	1.38E-11	0.526	2.02E-36	0.533	1.27E-39	0.371	2.52E-17	0.373	3.23E-18
Natural killer cell	KIR2DL1	0.018	5.66E-01	0.012	6.91E-01	0.103	2.19E-02	0.073	9.52E-02	−0.069	1.29E-01	−0.071	1.11E-01
Natural killer cell	KIR2DL3	0.049	1.19E-01	0.041	1.74E-01	0.079	8.17E-02	0.062	1.56E-01	−0.051	2.58E-01	−0.044	3.25E-01
Natural killer cell	KIR2DL4	0.031	3.28E-01	0.001	9.75E-01	0.071	1.14E-01	0.031	4.86E-01	0.079	8.12E-02	0.09	4.18E-02
Natural killer cell	KIR3DL1	0.05	1.12E-01	0.042	1.60E-01	0.108	1.65E-02	0.083	5.88E-02	0.016	7.21E-01	0.017	7.10E-01
Natural killer cell	KIR3DL2	0.028	3.74E-01	0.012	6.80E-01	0.2	7.96E-06	0.178	4.22E-05	−0.037	4.09E-01	−0.022	6.22E-01
Natural killer cell	KIR3DL3	0.004	9.06E-01	0	9.95E-01	0.053	2.41E-01	0.044	3.18E-01	−0.096	3.42E-02	−0.084	5.71E-02
Natural killer cell	KIR2DS4	0.015	6.43E-01	0.002	9.35E-01	0.042	3.55E-01	0.013	7.59E-01	−0.013	7.78E-01	−0.016	7.25E-01
Dendritic cell	HLA-DPB1	−0.038	2.33E-01	−0.044	1.41E-01	0.121	7.29E-03	0.093	3.38E-02	−0.225	5.19E-07	−0.211	1.50E-06
Dendritic cell	HLA-DQB1	−0.043	1.74E-01	−0.056	6.51E-02	0.067	1.37E-01	0.059	1.76E-01	−0.206	4.56E-06	−0.201	4.88E-06
Dendritic cell	HLA-DRA	0.156	8.14E-07	0.12	7.09E-05	0.172	1.22E-04	0.142	1.16E-03	−0.146	1.23E-03	−0.129	3.47E-03
Dendritic cell	HLA-DPA1	0.175	3.03E-08	0.134	8.84E-06	0.175	9.79E-05	0.151	5.55E-04	−0.196	1.32E-05	−0.173	8.73E-05
Dendritic cell	BDCA-1 (CD1C)	0.025	4.37E-01	0.006	8.52E-01	0.25	2.01E-08	0.223	2.59E-07	−0.18	6.44E-05	−0.162	2.40E-04
Dendritic cell	BDCA-4 (NRP1)	0.358	2.07E-31	0.334	4.34E-30	0.319	4.08E-13	0.29	1.40E-11	0.372	1.66E-17	0.371	4.84E-18
Dendritic cell	CD11c (ITGAX)	0.123	1.05E-04	0.088	3.61E-03	0.373	1.02E-17	0.306	9.03E-13	−0.144	1.38E-03	−0.127	3.99E-03
Dendritic cell	NKp46 (NCR1)	0.171	5.31E-08	0.161	8.41E-08	0.324	1.86E-13	0.312	2.76E-13	0.146	1.21E-03	0.146	9.71E-04

*BRCA, breast invasive carcinoma; HNSC, head and neck squamous cell carcinoma; THCA, thyroid carcinoma*.

Similarly, as shown in Table 3, for the adaptive immune cells, PERK had a significant association with the markers of Tfh (BCL6, IL21, and CD278) and Treg (FOXP3, CCR8, STST5B, and CD25) in BRCA (*p* < 0.05); CD8^+^ T cells (CD8A and CD8B), B cells (CD19, CD20, CD138, and CD23), Tfh (BCL6, IL21, CD278, and CXCL13), Th17 (STAT3 and IL17A), and Treg (FOXP3, CCR8, STST5B, TGFβ, and CD25) in HNSC (*p* < 0.05); and T cells (CD3D, CD3E, and CD2), Th1 (STAT4, STAT1, IFN-γ, and TNF-α), and T-cell exhaustion (PD-1, CTLA4, LAG3, and GZMB) in THCA (*p* < 0.05). Therefore, these results further confirmed the correlation between PERK and infiltrating immune cells in the microenvironment of BRCA, HNSC, and THCA. We speculated that the reason why PERK was highly expressed and was related to the poor prognosis in numerous cancers may be that it promoted significantly to the process of tumor immune escape.

**Table 3:** Correlation analysis between PERK and related gene markers of adaptive immune cells in TIMER in BRCA, HNSC, and THCA.

**Description**	**Gene markers**	**BRCA (*****n*** **=** **1093)**				**HNSC (*****n*** **=** **520)**				**THCA (*****n*** **=** **501)**			
		**Purity**		**None**		**Purity**		**None**		**Purity**		**None**	
		**Cor**	***p***	**Cor**	***P***	**Cor**	***p***	**Cor**	***p***	**Cor**	***p***	**Cor**	***P***
CD8+ T cell	CD8A	0.097	2.12E-03	0.057	5.81E-02	0.121	7.26E-03	0.089	4.13E-02	0.003	9.54E-01	0.015	7.33E-01
CD8+ T cell	CD8B	0.004	8.87E-01	−0.028	3.58E-01	0.096	3.24E-02	0.077	8.01E-02	−0.05	2.74E-01	−0.049	2.74E-01
T cell (general)	CD3D	−0.056	7.79E-02	−0.079	8.65E-03	0.067	1.39E-01	0.043	3.25E-01	−0.211	2.46E-06	−0.199	5.84E-06
T cell (general)	CD3E	0.012	7.00E-01	−0.024	4.23E-01	0.204	5.34E-06	0.167	1.27E-04	−0.156	5.27E-04	−0.146	9.66E-04
T cell (general)	CD2	0.062	5.09E-02	0.021	4.96E-01	0.156	5.19E-04	0.128	3.32E-03	−0.173	1.27E-04	−0.158	3.36E-04
B cell	CD19	−0.062	5.22E-02	−0.079	8.94E-03	0.348	1.83E-15	0.309	5.67E-13	−0.104	2.20E-02	−0.086	5.15E-02
B cell	CD20 (MS4A1)	0.065	4.06E-02	0.026	3.92E-01	0.383	1.19E-18	0.328	1.39E-14	0	9.91E-01	0.012	7.84E-01
B cell	CD138 (SDC1)	−0.004	9.08E-01	−0.007	8.10E-01	0.207	3.77E-06	0.201	3.85E-06	0.134	3.07E-03	0.135	2.27E-03
B cell	CD23 (FCER2)	−0.096	2.39E-03	−0.105	4.82E-04	0.345	3.59E-15	0.312	2.75E-13	−0.132	3.55E-03	−0.125	4.67E-03
Th1	T-bet (TBX21)	0.021	5.01E-01	−0.012	6.91E-01	0.136	2.49E-03	0.109	1.24E-02	−0.072	1.14E-01	−0.059	1.83E-01
Th1	STAT4	0.15	1.98E-06	0.104	5.59E-04	0.235	1.42E-07	0.205	2.37E-06	−0.193	1.68E-05	−0.186	2.43E-05
Th1	STAT1	0.354	1.14E-30	0.341	2.42E-31	0.063	1.63E-01	0.041	3.48E-01	0.144	1.44E-03	0.162	2.51E-04
Th1	IFN-γ (IFNG)	0.083	8.53E-03	0.047	1.22E-01	−0.033	4.70E-01	−0.05	2.55E-01	−0.115	1.13E-02	−0.105	1.76E-02
Th1	TNF-α (TNF)	−0.002	9.54E-01	−0.018	5.41E-01	0.143	1.52E-03	0.116	7.91E-03	−0.167	2.12E-04	−0.154	5.10E-04
Th2	GATA3	0.159	4.97E-07	0.181	1.50E-09	0.073	1.08E-01	0.051	2.43E-01	0.062	1.72E-01	0.08	7.27E-02
Th2	STAT6	0.207	4.76E-11	0.21	1.99E-12	0.414	8.53E-22	0.427	0.00E+00	0.235	1.54E-07	0.242	3.14E-08
Th2	STAT5A	0.022	4.91E-01	0.025	4.08E-01	0.298	1.60E-11	0.29	1.49E-11	0.03	5.03E-01	0.034	4.40E-01
Th2	IL13	−0.022	4.98E-01	−0.036	2.31E-01	0.068	1.30E-01	0.054	2.16E-01	−0.128	4.75E-03	−0.124	5.11E-03
Tfh	BCL6	0.208	3.72E-11	0.201	1.82E-11	0.68	6.19E-68	0.686	0.00E+00	0.213	1.94E-06	0.215	9.49E-07
Tfh	IL21	0.129	4.53E-05	0.115	1.31E-04	0.209	3.02E-06	0.213	8.57E-07	0.005	9.19E-01	0.013	7.78E-01
Tfh	CD278 (ICOS)	0.126	6.88E-05	0.084	5.14E-03	0.197	1.12E-05	0.165	1.57E-04	−0.083	6.58E-02	−0.066	1.37E-01
Tfh	CXCL13	−0.035	2.64E-01	−0.058	5.36E-02	0.256	8.63E-09	0.205	2.48E-06	−0.067	1.40E-01	−0.063	1.59E-01
Th17	STAT3	0.437	1.45E-47	0.454	6.26E-57	0.567	3.19E-43	0.572	0.00E+00	0.539	4.19E-38	0.542	2.97E-40
Th17	IL17A	0.02	5.24E-01	0.024	4.34E-01	0.191	1.99E-05	0.178	4.43E-05	−0.074	1.04E-01	−0.066	1.38E-01
Treg	FOXP3	0.082	9.84E-03	0.044	1.47E-01	0.37	2.11E-17	0.331	1.00E-14	−0.181	5.69E-05	−0.159	3.14E-04
Treg	CCR8	0.275	9.54E-19	0.242	3.91E-16	0.492	2.13E-31	0.453	9.81E-28	0.06	1.89E-01	0.073	9.89E-02
Treg	STAT5B	0.237	3.84E-14	0.246	1.20E-16	0.541	8.22E-39	0.536	3.54E-40	0.523	1.18E-35	0.532	1.47E-38
Treg	TGFβ (TGFB1)	0.014	6.59E-01	0.01	7.36E-01	0.102	2.40E-02	0.069	1.14E-01	0.055	2.27E-01	0.048	2.84E-01
Treg	CD25 (IL2RA)	0.167	1.13E-07	0.12	7.00E-05	0.369	2.80E-17	0.316	1.87E-13	0.019	6.71E-01	0.028	5.28E-01
T-cell exhaustion	PD-1 (PDCD1)	−0.059	6.12E-02	−0.083	6.06E-03	0.113	1.20E-02	0.086	4.86E-02	−0.124	5.91E-03	−0.106	1.68E-02
T-cell exhaustion	CTLA4	0.012	7.04E-01	−0.021	4.96E-01	0.121	7.39E-03	0.088	4.51E-02	−0.188	2.94E-05	−0.173	8.54E-05
T-cell exhaustion	LAG3	−0.089	4.88E-03	−0.116	1.09E-04	−0.013	7.81E-01	−0.033	4.47E-01	−0.208	3.49E-06	−0.203	3.75E-06
T-cell exhaustion	TIM-3 (HAVCR2)	0.224	8.53E-13	0.194	7.79E-11	0.215	1.57E-06	0.179	4.13E-05	−0.071	1.16E-01	−0.059	1.84E-01
T-cell exhaustion	GZMB	−0.044	1.70E-01	−0.065	3.08E-02	−0.019	6.74E-01	−0.045	3.08E-01	−0.193	1.81E-05	−0.187	2.08E-05

## Discussion

Previous studies have proven that PERK has the function of supporting tumor growth, metastasis, autophagy, and radiation resistance (Bobrovnikova-Marjon et al., 2010; Avivar-Valderas et al., 2011; Rouschop et al., 2013; Liu et al., 2015; Salaroglio et al., 2017). Herein, we report the correlation between PERK expression level and the prognosis of various cancers. The high-expression level of PERK correlates with a poorer prognosis in KIRP, LGG, BRCA, and THCA and with a favorable prognosis in HNSC. Moreover, our analyses show that in BRCA, HNSC, and THCA, the immune infiltration levels and diverse immune marker sets are correlated with the levels of PERK expression. Thus, our study provides insights into understanding the potential role of PERK in tumor immunology and its potential application as a cancer biomarker.

In this study, we examined the expression levels of PERK and the systematic prognostic landscape in different types of cancers. The differential expression of PERK between cancer and normal tissues was observed in many types of cancers. Based on the Oncomine database, we found that PERK was upregulated in brain and CNS cancer, head and neck cancer, and breast cancer and downregulated in lymphoma and sarcoma (Figure 2A). However, in the TIMER database, the expression of PERK mRNA was upregulated in BRCA, CHOL, LIHC, LUAD, LUSC, and STAD and downregulated in COAD, READ, and THCA, compared with normal adjacent tissues (Figure 2B). Nevertheless, in these databases, we found consistent prognostic correlations between PERK expression in KIRP, LGG, BRCA, THCA, and HNSC. The PrognoScan database search revealed that increased PERK expression correlated with poor prognosis in brain cancer and soft tissue cancer. Lung cancer was an exception where high levels of PERK expression showed a better prognosis. Furthermore, data analysis from the Kaplan–Meier plotter showed that a high level of PERK expression was correlated with favorable prognosis in bladder carcinoma, esophageal squamous cell carcinoma, lung adenocarcinoma, rectum adenocarcinoma, and thymoma and related to a poor prognosis in kidney renal papillary cell carcinoma, liver hepatocellular carcinoma, and thyroid carcinoma (Figure 4). The UALCAN database analysis demonstrated that the high expression of PERK was associated with a poor prognosis in KIRP, LGG, THCA, and BRCA (Figure 5). These results are consistent with previous findings that PERK signaling in cancer contributes to adaptive pathways rather than to cancer cell death, as demonstrated by the fact that pharmacologic inhibition of PERK attenuates tumor growth (Wang et al., 2017, 2019; Bagratuni et al., 2020). PERK's prometastatic functions in breast cancer were mediated by its downstream transcription factor CREB3L1. Inhibition of CREB3L1 by genetic or pharmacological methods suppresses cancer cell invasion and metastasis (Feng et al., 2017). Another report showed that inhibition of the PERK–eIF2α-GRP94 signaling pathway silenced the epidermal growth factor receptor (EGFR) and then increased the radiosensitivity of both radiosensitive and radioresistant oropharyngeal squamous cell carcinoma (OSCC) cells (Zhang et al., 2018). Also, PERK's expression increased the expression of phosphorylated eIF2α (p-eIF2α) and promoted G0–G1 arrest and survival of cancer cells *in vitro*, suggesting that eIF2α phosphorylation can initiate cytoprotective effects (Ranganathan et al., 2008). Taken together, these findings strongly suggest that PERK is a prognostic biomarker in BRCA, HNSC, and THCA.

We then investigated the potential functions of PERK in certain cancers and found that PERK was significantly enriched in the immune-related signaling pathways in BRCA, HNSC, and THCA, including B-cell differentiation, T-cell differentiation, T-cell activation, etc. This finding is consistent with previous studies showing that PERK has an essential function in the differentiation of naive B cells into plasma cells (Gass et al., 2008; Ma et al., 2010; Zhu et al., 2019). PERK has also been reported to play an important role in T-cell development, albeit in an ATF4-independent manner (Solanki et al., 2016). Further analysis demonstrated the strong associations between PERK expression and tumor-infiltrating immune cells, especially B cells, CD8^+^ T cells, macrophages, neutrophils, and dendritic cells, in BRCA, HNSC, and THCA (Figure 7). Moreover, the correlation between PERK expression and the marker genes of immune cells implicates the role of PERK in regulating tumor immunology in BRCA, HNSC, and THCA. Gene markers of M1 macrophages such as INOS and CXCL10 and M2 macrophage markers such as CD163 and IL10 showed strong correlations with PERK expression (Table 2). These results prove that PERK could regulate the polarization of tumor-associated macrophages. Similarly, PERK's function in regulating the phenotypic polarization of macrophages has been reported during the pathological progression of nonalcoholic fatty liver disease (NAFLD) (Yang et al., 2019). Additionally, our results also indicated that PERK is capable of activating Tregs and inducing T-cell exhaustion. The elevation of PERK expression positively correlates with the expression of Treg and T-cell exhaustion marker genes (FOXP3, CCR8, STST5B, CD25PD-1, CTLA4, LAG3, and GZMB in HNSC and THCA, Table 3). These results are consistent with a recent study revealing that the PERK signaling pathway contributes to mitochondrial exhaustion of T effector cells (Hurst et al., 2019). Furthermore, significant correlations were observed between PERK expression and the regulation of several markers of T-helper cells (Th17 and Th1) in HNSC and THCA. These correlations suggested a potential mechanism where PERK regulates T-cell functions in these cancers. In sum, our results initially indicate that PERK plays critical roles in recruiting infiltrating immune cells and attenuating the tumor immune system in BRCA, HNSC, and THCA.

Although there are few studies reporting the function of PERK in tumor immunoregulation, a recent report provides a possible mechanism which explains why PERK expression correlates with immune infiltration and poor prognosis. The myelopoiesis process that protects against tumors is drastically damaged in most cancers that block protective antitumor T-cell immunity and promote cancer cell progression (Singhal et al., 2016). Expansion of myeloid-derived suppressor cells (MDSCs) has emerged as a key mechanism of antitumor immune evasion and correlates with a poor clinical outcome and resistance to cancer immunotherapy (Lu et al., 2017). It was determined that PERK signaling increased in tumor-infiltrating MDSCs, mediating the immunosuppression pathway in tumors through inhibition of STING signaling. PERK deletion transformed MDSCs into myeloid cells that activated CD8^+^ T-cell-mediated immunity against cancer (Mohamed et al., 2020). Another study showed that inhibition of PERK in CD8^+^ T cells abrogates mitochondrial ROS generation in PD-1^+^ CD8^+^ tumor-infiltrating lymphocytes (TILs), which boosts CD8^+^ TIL viability and enhances antitumor immunity (Hurst et al., 2019). Therefore, PERK seems to be expressed predominantly in immune cells rather than cancer cells, and its function relates to promote tumor immune escape in BRCA, HNSC, and THCA.

## Conclusion

In this study, we found that increased PERK expression correlates with poor prognosis and increased immune infiltration levels in B cells, CD8^+^ T cells, macrophages, neutrophils, and dendritic cells of multiple cancers, especially in breast and thyroid cancers. In addition, PERK expression potentially contributes to the regulation of tumor-associated macrophages, T-cell exhaustion, and Tregs. Therefore, PERK likely plays a pivotal role in immune cell infiltration and as a prognosis biomarker in patients with BRCA, HNSC, and THCA. However, our study lacks the *in vitro* and animal experiments to confirm the role of PERK in the growth and progression of BRCA, HNSC, and THCA and its relationship with the infiltration of immune cells in the tumor microenvironment. Therefore, further research is needed to verify the role of PERK in cancers using these models.

## Data Availability Statement

The datasets presented in this study can be found in online repositories. The names of the repository/repositories and accession number(s) can be found in the article/Supplementary Material.

## Author Contributions

PW and GZ designed this study, analyzed the data, and supervised the entire study. PW, LH, MY, XL, and YS extracted the information from the databases. PW wrote the manuscript. GZ revised the manuscript. All authors contributed to the article and approved the submitted version.

## Conflict of Interest

The authors declare that the research was conducted in the absence of any commercial or financial relationships that could be construed as a potential conflict of interest.

## Abbreviations

PERK: protein kinase R (PKR)-like endoplasmic reticulum kinase
EIF2AK3: eukaryotic translation initiation factor 2 alpha kinase 3
TCGA: The Cancer Genome Atlas
ACC: adrenocortical carcinoma
BLCA: bladder urothelial carcinoma
BRCA: breast invasive carcinoma
CESC: cervical squamous cell carcinoma and endocervical adenocarcinoma
CHOL: cholangiocarcinoma
COAD: colon adenocarcinoma
DLBC: lymphoid neoplasm diffuse large B-cell lymphoma
ESCA: esophageal carcinoma
GBM: glioblastoma multiforme
HNSC: head and neck squamous cell carcinoma
KICH: kidney chromophobe
KIRC: kidney renal clear cell carcinoma
KIRP: kidney renal papillary cell carcinoma
LAML: acute myeloid leukemia
LGG: brain lower grade glioma
LIHC: liver hepatocellular carcinoma
LUAD: lung adenocarcinoma
LUSC: lung squamous cell carcinoma
MESO: mesothelioma
OV: ovarian serous cystadenocarcinoma
PAAD: pancreatic adenocarcinoma
PCPG: pheochromocytoma and paraganglioma
PRAD: prostate adenocarcinoma
READ: rectum adenocarcinoma
SARC: sarcoma
SKCM: skin cutaneous melanoma
STAD: stomach adenocarcinoma
TGCT: testicular germ cell tumors
THCA: thyroid carcinoma
THYM: thymoma
UCEC: uterine corpus endometrial carcinoma
UCS: uterine carcinosarcoma
UVM: uveal melanoma.
