# Supplementary figures and images for: The Prognostic Value of PERK in Cancer and Its Relationship With Immune Cell Infiltration
Source: Front Mol Biosci. 2021 Apr 16;8:648752. doi: 10.3389/fmolb.2021.648752 (PMC8085429; doi:10.3389/fmolb.2021.648752)

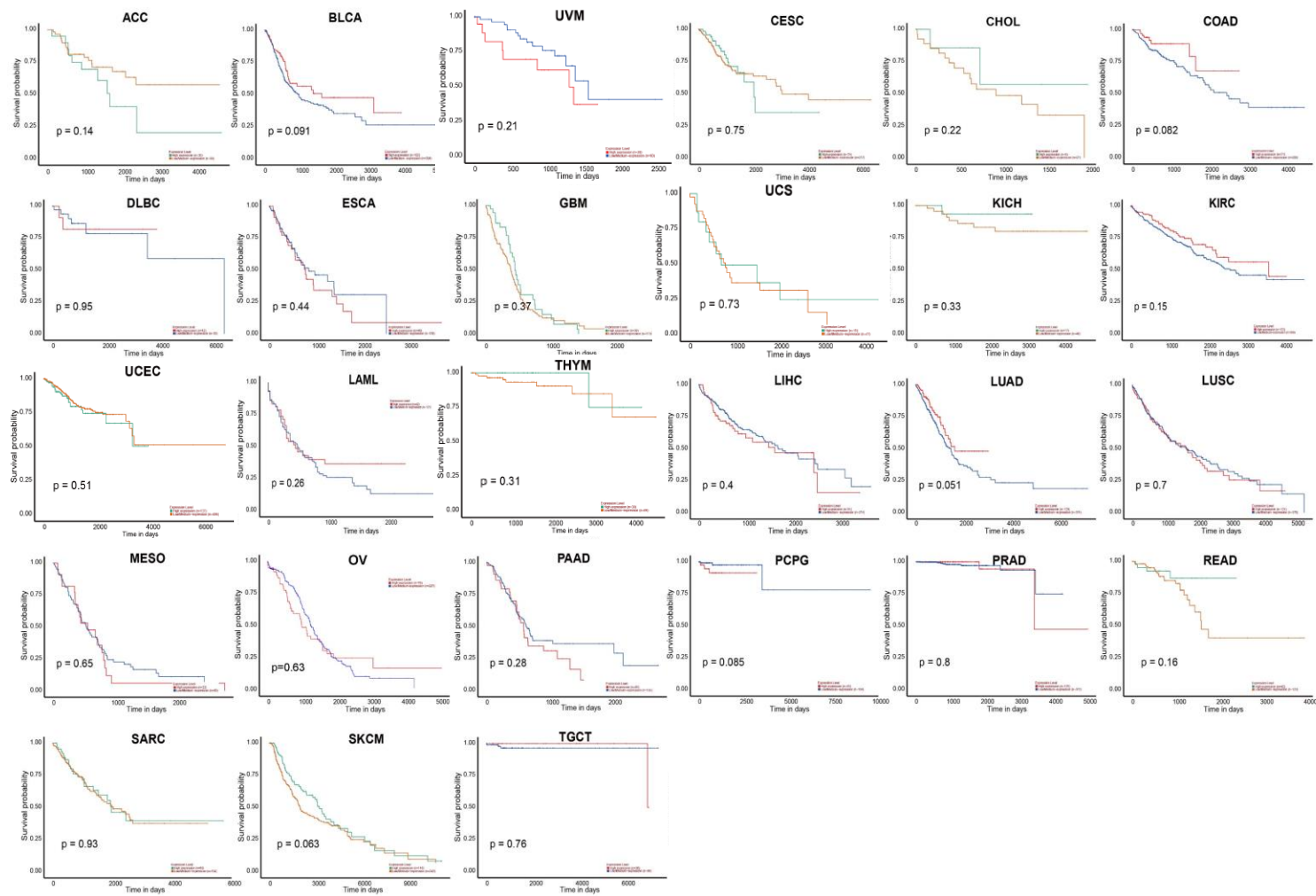

Supplement: Supplementary file 13 [file Image_1.PDF]

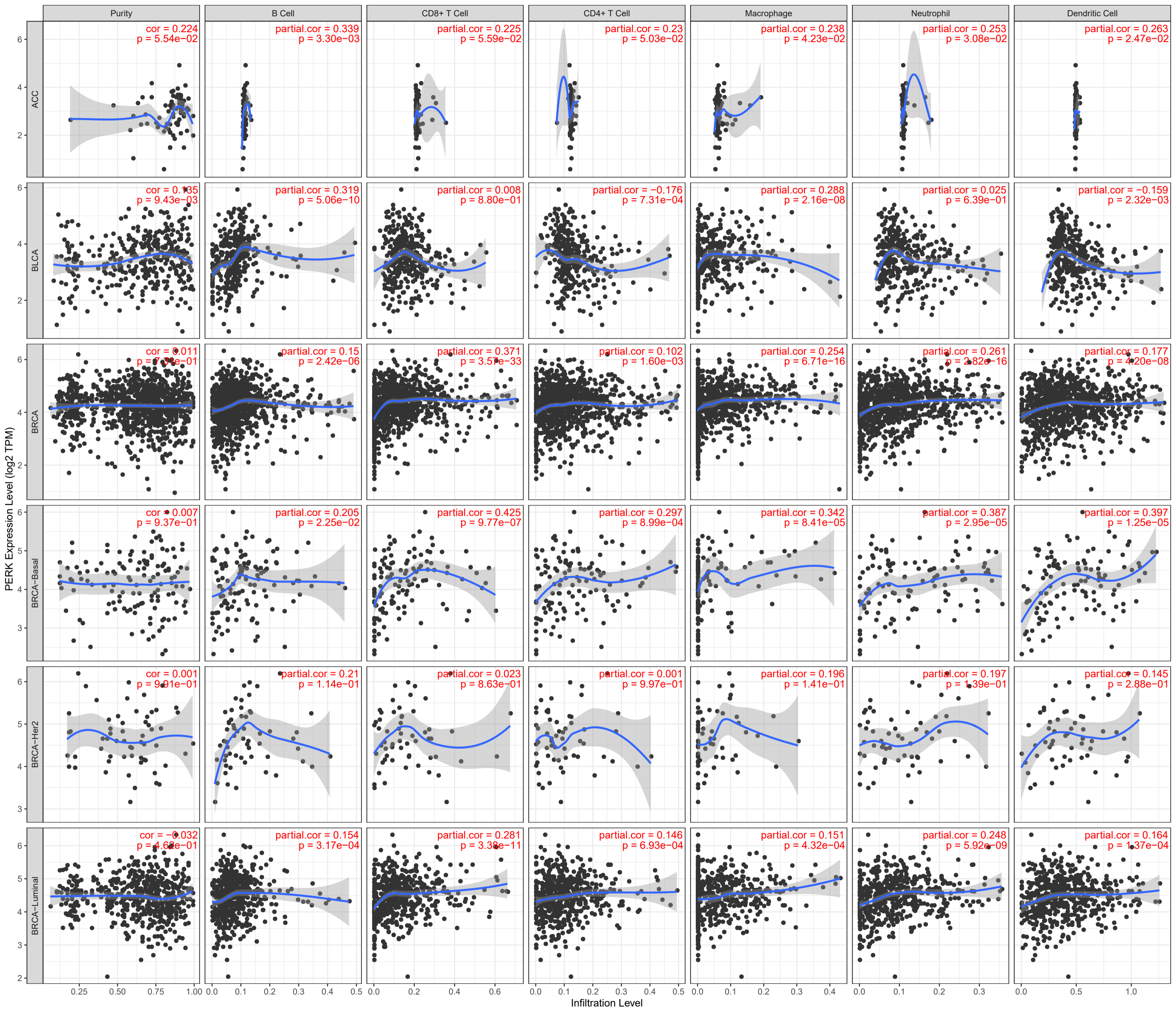

Supplement: Supplementary file 14 [file Image_2.PDF]

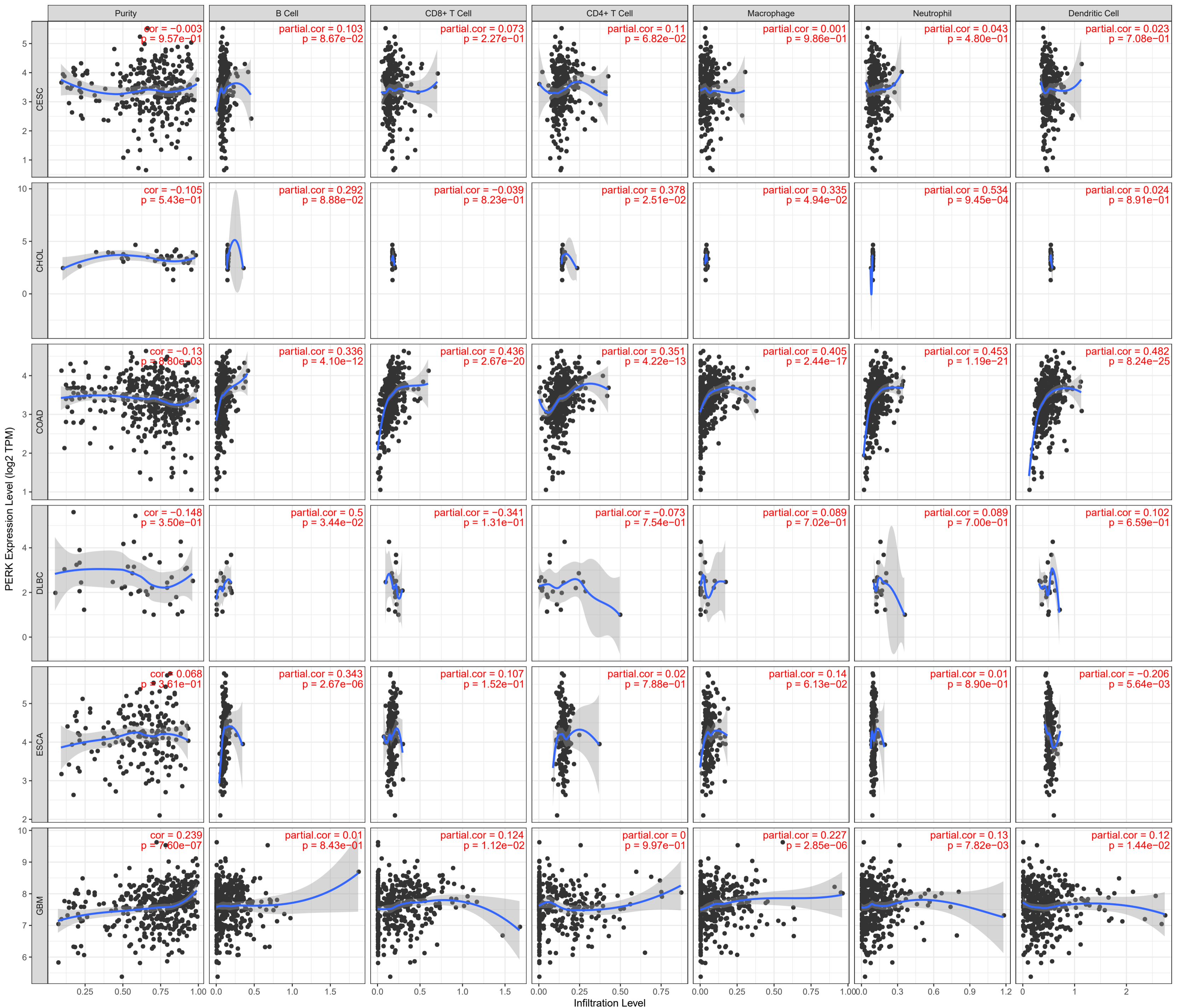

Supplement: Supplementary file 15 [file Image_3.PDF]

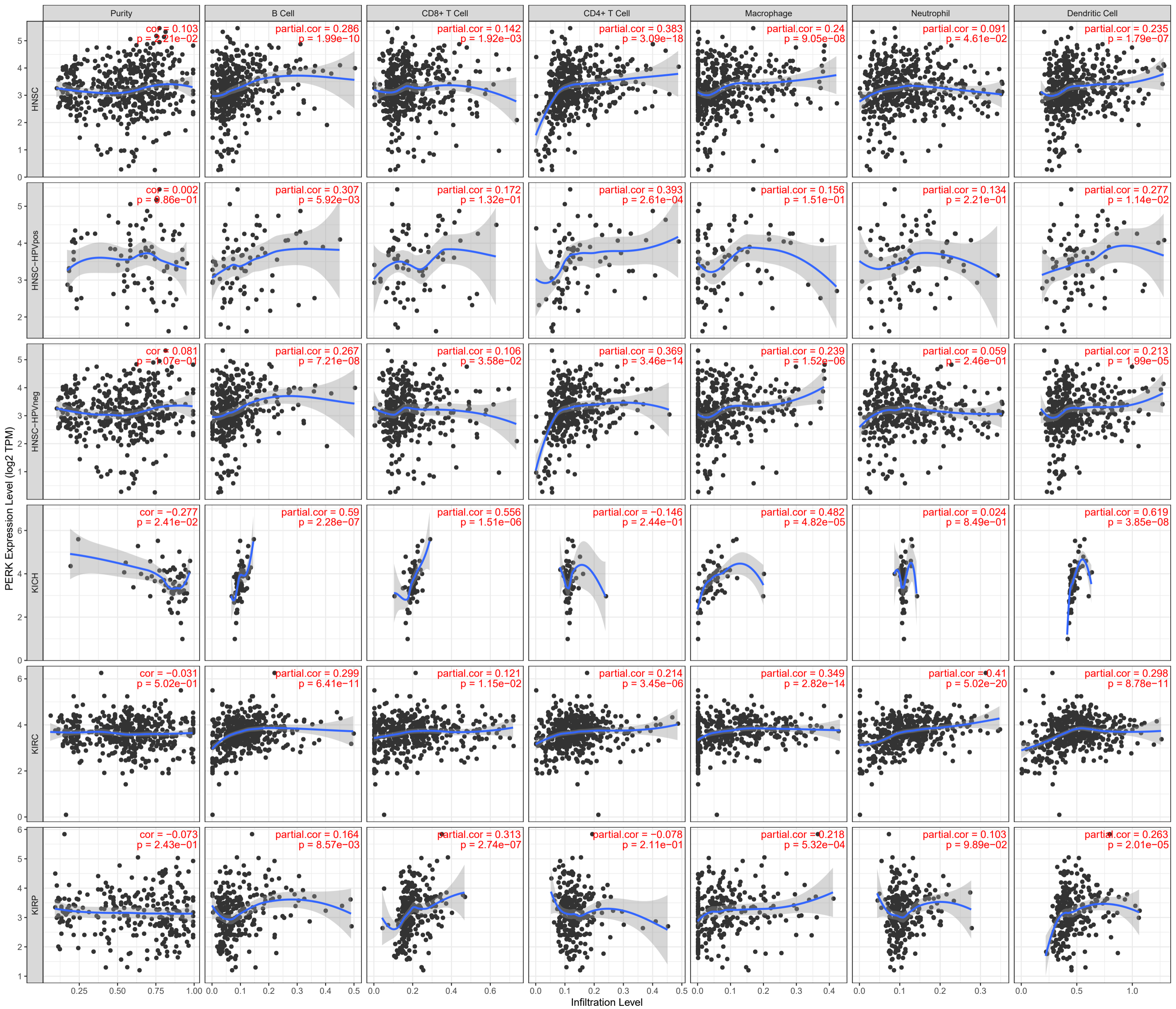

Supplement: Supplementary file 16 [file Image_4.PDF]

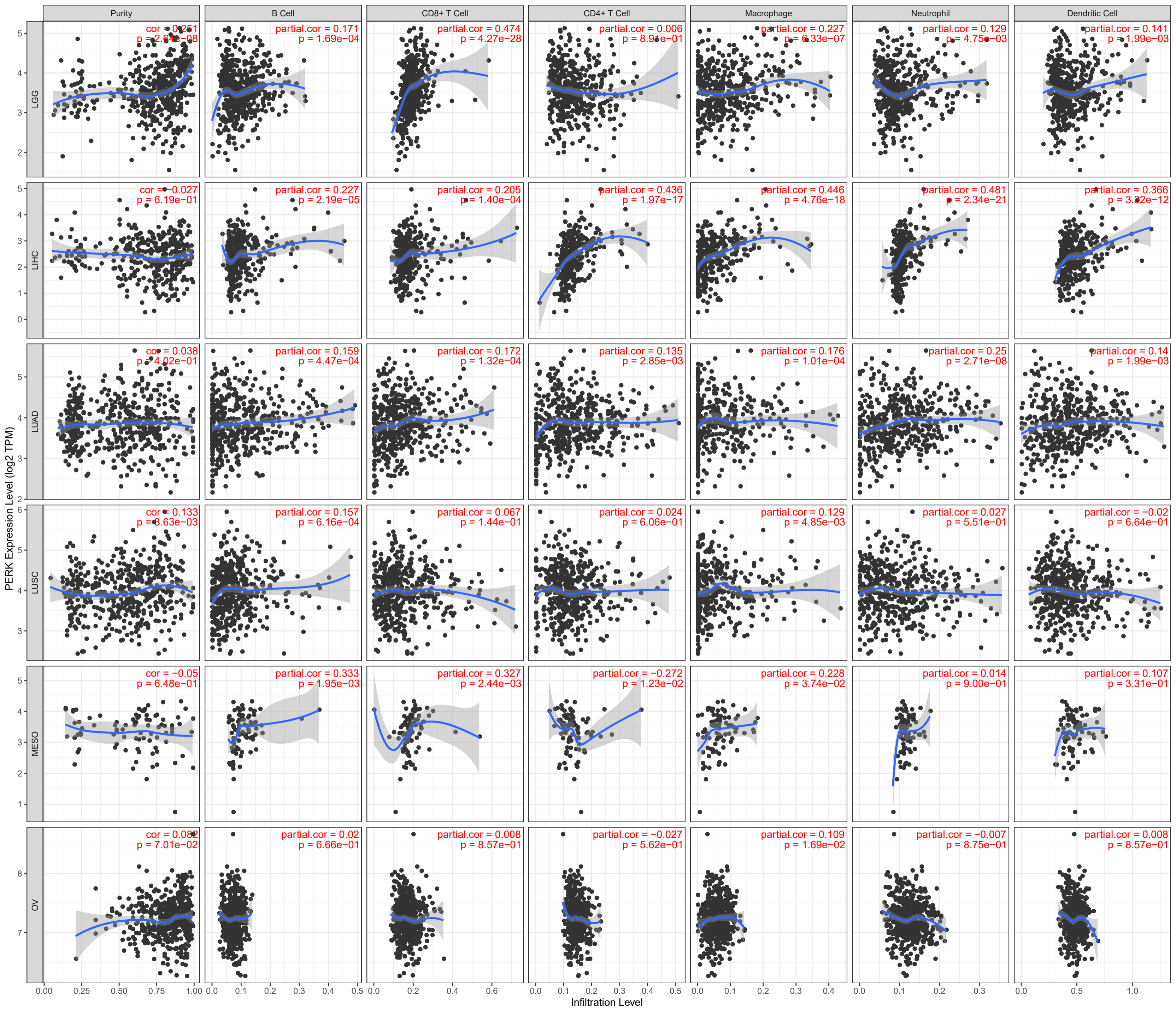

Supplement: Supplementary file 17 [file Image_5.PDF]

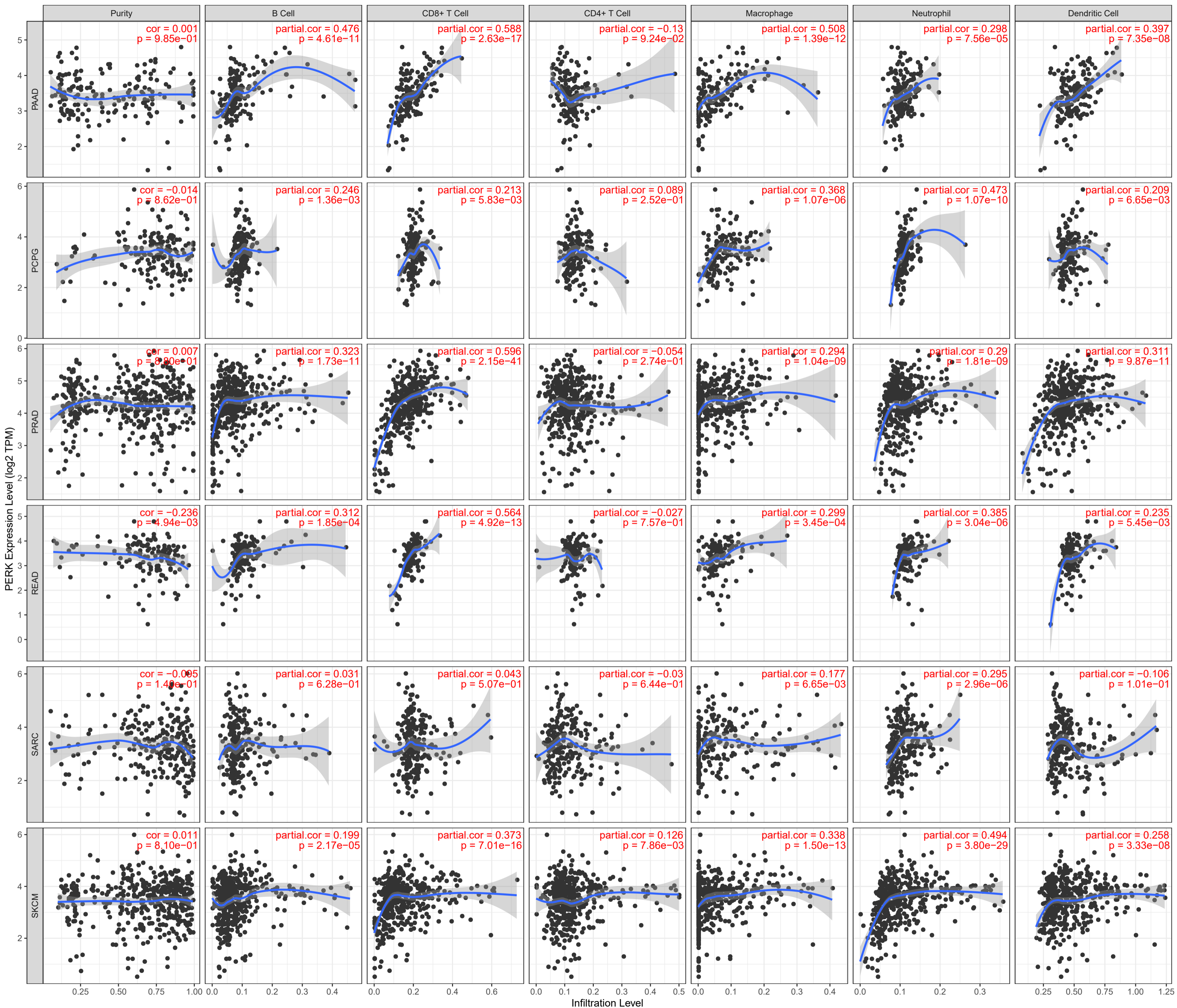

Supplement: Supplementary file 18 [file Image_6.PDF]

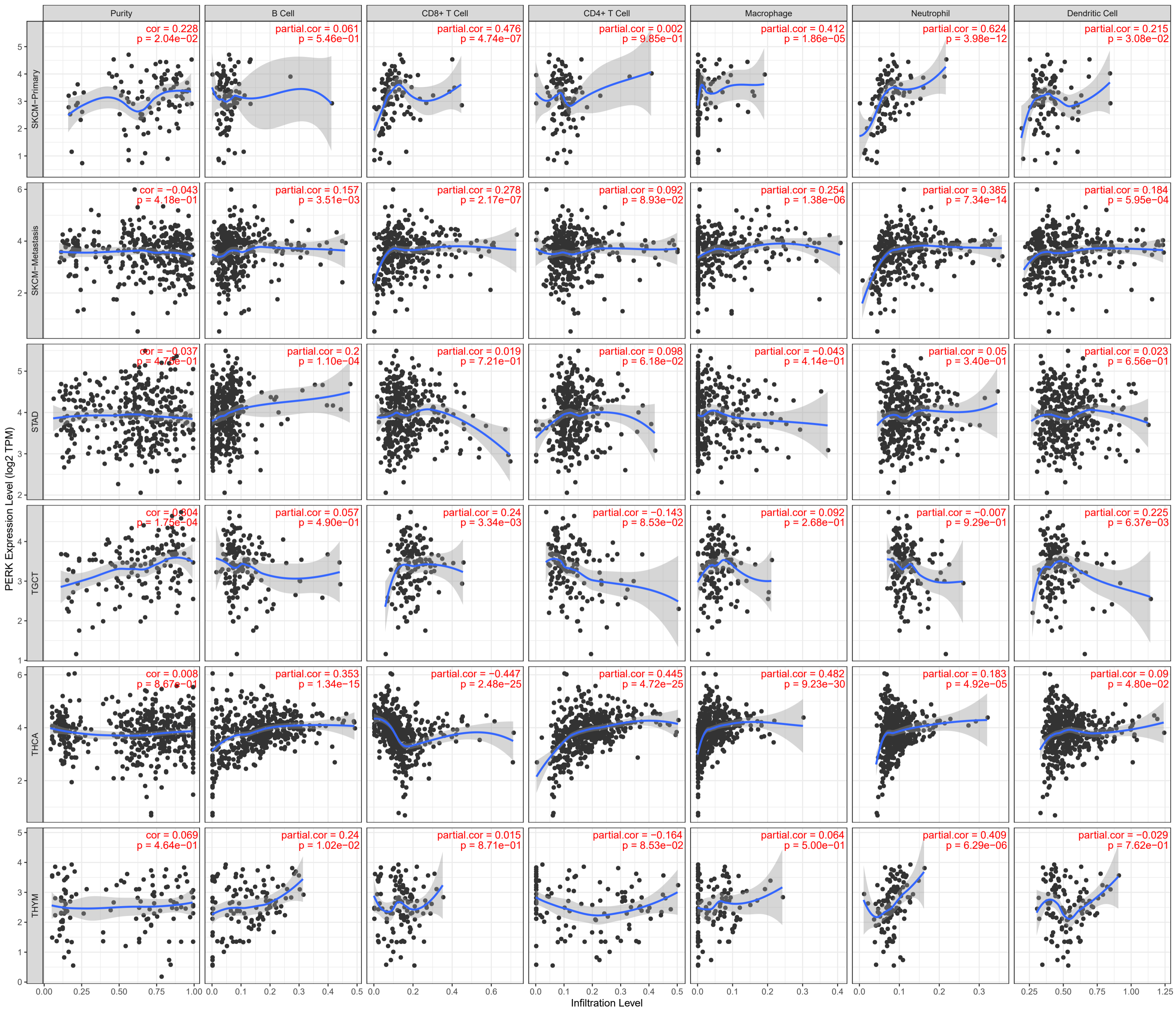

Supplement: Supplementary file 19 [file Image_7.PDF]

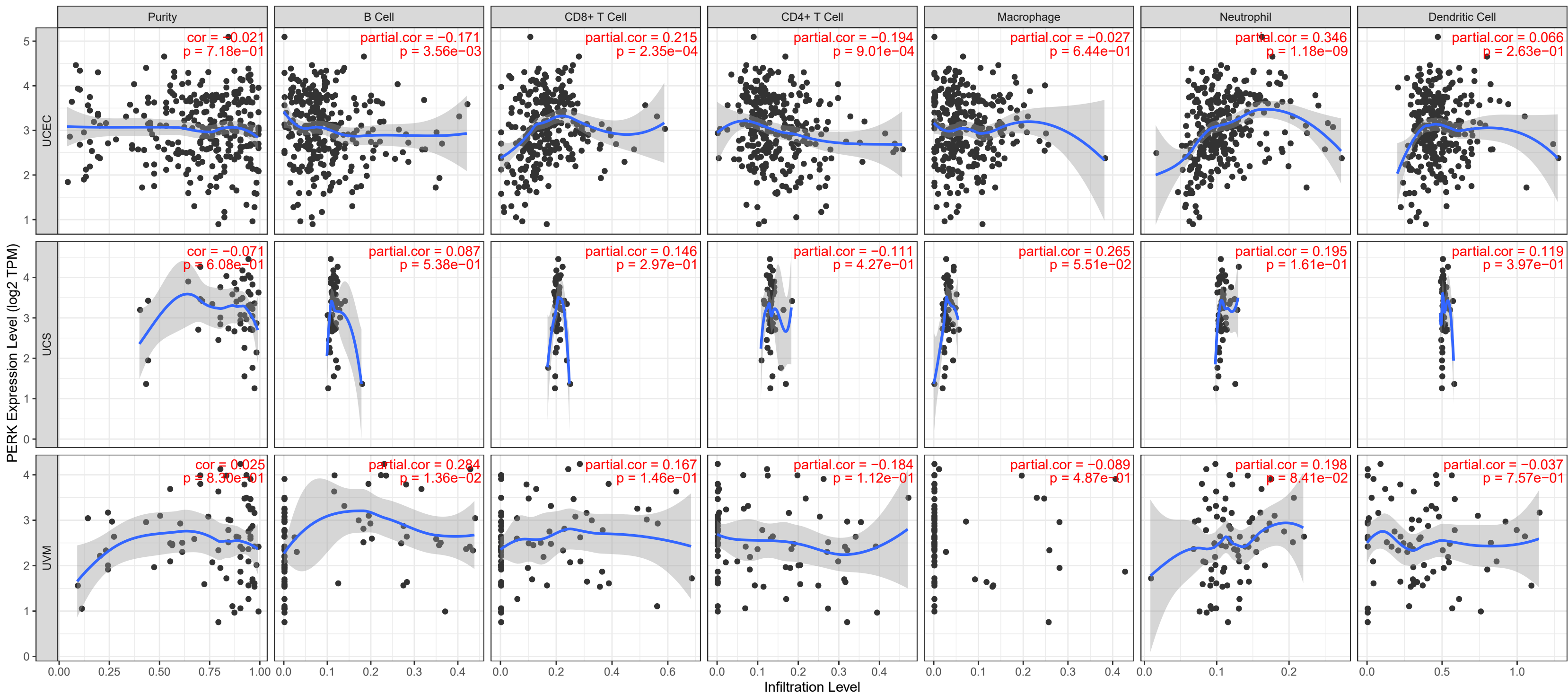

Supplement: Supplementary file 20 [file Image_8.PDF]
